# Obstetric Volume and Severe Maternal Morbidity Among Low-Risk and Higher-Risk Patients Giving Birth at Rural and Urban US Hospitals

**DOI:** 10.1001/jamahealthforum.2023.2110

**Published:** 2023-06-24

**Authors:** Katy Backes Kozhimannil, Stephanie A. Leonard, Sara C. Handley, Molly Passarella, Elliott K. Main, Scott A. Lorch, Ciaran S. Phibbs

**Affiliations:** 1 Division of Health Policy and Management, University of Minnesota School of Public Health, Minneapolis; 2 Department of Obstetrics & Gynecology, Stanford University School of Medicine, Stanford, California; 3 California Maternal Quality Care Collaborative, Stanford; 4 Department of Pediatrics, Children’s Hospital of Philadelphia, Philadelphia, Pennsylvania; 5 Leonard Davis Institute of Health Economics, Wharton School, University of Pennsylvania, Philadelphia; 6 Health Economics Resource Center, Veterans Affairs Palo Alto Healthcare System, Menlo Park, California; 7 Departments of Pediatrics and Health Policy, Stanford University School of Medicine, Stanford, California

## Abstract

**Importance:**

Identifying hospital factors associated with severe maternal morbidity (SMM) is essential to clinical and policy efforts.

**Objective:**

To assess associations between obstetric volume and SMM in rural and urban hospitals and examine whether these associations differ for low-risk and higher-risk patients.

**Design, Setting, and Participants:**

This retrospective cross-sectional study of linked vital statistics and patient discharge data was conducted from 2022 to 2023. Live births and stillbirths (≥20 weeks’ gestation) at hospitals in California (2004-2018), Michigan (2004-2020), Pennsylvania (2004-2014), and South Carolina (2004-2020) were included. Data were analyzed from December 2022 to May 2023.

**Exposures:**

Annual birth volume categories (low, medium, medium-high, and high) for hospitals in urban (10-500, 501-1000, 1001-2000, and >2000) and rural (10-110, 111-240, 241-460, and >460) counties.

**Main Outcome and Measures:**

The main outcome was SMM (excluding blood transfusion); covariates included age, payer status, educational attainment, race and ethnicity, and obstetric comorbidities. Analyses were stratified for low-risk and higher-risk obstetric patients based on presence of at least 1 clinical comorbidity.

**Results:**

Among more than 11 million urban births and 519 953 rural births, rates of SMM ranged from 0.73% to 0.50% across urban hospital volume categories (high to low) and from 0.47% to 0.70% across rural hospital volume categories (high to low). Risk of SMM was elevated for patients who gave birth at rural hospitals with annual birth volume of 10 to 110 (adjusted risk ratio [ARR], 1.65; 95% CI, 1.14-2.39), 111 to 240 (ARR, 1.37; 95% CI, 1.10-1.70), and 241 to 460 (ARR, 1.26; 95% CI, 1.05-1.51), compared with rural hospitals with greater than 460 births. Increased risk of SMM occurred for low-risk and higher-risk obstetric patients who delivered at rural hospitals with lower birth volumes, with low-risk rural patients having notable discrepancies in SMM risk between low (ARR, 2.32; 95% CI, 1.32-4.07), medium (ARR, 1.66; 95% CI, 1.20-2.28), and medium-high (ARR, 1.68; 95% CI, 1.29-2.18) volume hospitals compared with high volume (>460 births) rural hospitals. Among hospitals in urban counties, there was no significant association between birth volume and SMM for low-risk or higher-risk obstetric patients.

**Conclusions and Relevance:**

In this cross-sectional study of births in US rural and urban counties, risk of SMM was elevated for low-risk and higher-risk obstetric patients who gave birth in lower-volume hospitals in rural counties, compared with similar patients who gave birth at rural hospitals with greater than 460 annual births. These findings imply a need for tailored quality improvement strategies for lower volume hospitals in rural communities.

## Key Points

**Question:**

What is the association between obstetric volume and severe maternal morbidity in US rural and urban hospitals, and do these associations differ for low-risk and higher-risk patients?

**Findings:**

In this cross-sectional study of more than 11 million births in urban counties and 519 953 births in rural counties, risk of severe maternal morbidity was elevated for low-risk and higher-risk obstetric patients who gave birth in lower-volume rural hospitals, compared with similar patients who gave birth at rural hospitals with more than 460 annual births. No significant volume-outcome association was detected among urban hospitals.

**Meaning:**

These findings imply a need for tailored quality improvement strategies for lower-volume hospitals in rural communities.

## Teaser

This cross-sectional study assesses associations between obstetric volume and severe maternal morbidity in rural and urban hospitals and examines whether these associations differ for low-risk and higher-risk patients.

## Introduction

Maternal mortality and morbidity rates in the US are far above those for other high-income countries, with US maternal mortality rates doubling since 1987^1^ and rising rates of maternal hemorrhage.^2^ One potential driver of maternal health is the characteristics of hospitals where patients give birth, including obstetric volume. There is a positive volume-outcome association for many surgical conditions, such as trauma care and complex surgical procedures,^3,4,5^ and for neonatal intensive care units,^6,7,8^ but there are less available data for obstetric services. Some evidence indicates that the volume of births is associated with maternal outcomes, with lower-volume facilities having higher risks of mortality and serious morbidity,^9,10^ although this evidence is not consistent for all maternal outcomes or across all types of rural/urban geographic locations.

Whether hospital obstetric volume may be associated with poor maternal health could have important policy implications. Given that the existing literature suggests some potential benefits of increased obstetric volume, there could be benefits from strategic consolidation of obstetric services in select settings.^9,10^ However, there are trade-offs between service consolidation and access. Currently, to our knowledge, there are no standards on appropriate birth volume thresholds or data on how such thresholds change when routine access to medical care is limited, such as in rural areas, or if there are differences for obstetric patients with varying levels of clinical risk, including low-risk patients.^11^

This study examined the associations between annual birth volume and severe maternal morbidity (SMM) in rural and urban US hospitals. We also examined whether the volume-outcome association differs for low-risk and higher-risk patients giving birth in rural and urban hospital settings in the US.

## Methods

This retrospective, cross-sectional study was reviewed and approved by the Institutional Review Board at Stanford University and the human research committees at each of the states that released data for this study. The need for informed consent was waived under 45 CFR 46 (the Common Rule). This study followed the Strengthening the Reporting of Observational Studies in Epidemiology (STROBE) reporting guideline.

### Setting and Participants

This analysis used linked vital statistics–patient discharge data, which comprised live birth and fetal death certificate records linked to maternal and infant patient-level hospital discharge data (including transfers and readmissions) from California (2004-2018), Michigan (2004-2020), Pennsylvania (2004-2014), and South Carolina (2004-2020). While not a nationally representative sample, these states make linked data available and reflect a diversity of geography (rural and urban areas), sociodemographic characteristics, and health care systems.

We identified hospitals based on their location and by metropolitan vs nonmetropolitan Urban Influence Codes. These codes are based on each county’s US Census Bureau Metropolitan Statistical Area designation.^1^ We categorized hospitals in metropolitan counties as urban, and hospitals located in nonmetropolitan counties as rural, including both micropolitan (rural county with population center of 10 000 to <50 000 people) and noncore (rural county with a population center of <10 000) rural counties.^12^ There are multiple methods for measuring rurality,^3^ and this definition was chosen based on availability of data elements, consistency with prior research, and policy and administrative relevance for rural communities, in particular.^4,5,6,7^

Each hospital was included during the years for which data were available in each state; a hospital-year is the number of observations of annual birth volume and SMM rates (per hospital, per year). This analysis included data on 11 023 423 births that occurred in 5846 hospital-years in urban counties, and 519 953 births in 1335 hospital-years in rural counties from 2004 to 2020 in 4 US states.

### Exposures

Hospital obstetric volume was determined by the total number of live births and stillbirths (≥20 weeks’ gestation) occurring during a calendar year; including stillbirths is important, as SMM rates are elevated for this population.^13^ We excluded records from hospitals that had fewer than 10 annual births for half or more of the years they appeared in the data.

Annual birth volume categories were created (low, medium, medium-high, and high) for hospitals located in urban and rural US counties. Replicating prior research that measured birth volume quartiles for rural and urban hospitals, the categories for urban counties were defined as low (10-500 births), medium (501-1000 births), medium-high (1001-2000 births), and high (>2000 births).^4^ The birth volume categories for rural counties were defined as low (10-110 births), medium (111-240 births), medium-high (241-460 births), and high (>460 births).^7^

To distinguish those with no clinical risk factors from those with 1 or more risk factors for SMM, analyses are stratified for low-risk and higher-risk obstetric patients based on the presence of at least 1 clinical comorbidity,^14,15^ measured by an enhanced obstetric comorbidity score that was developed and validated to improve comparisons of SMM rates across patient populations with different comorbidity case mixes. This index includes 27 comorbidities ranging in prevalence and weighted by severity in relation to SMM risk (ie, placenta accreta spectrum to advanced maternal age ≥35 years); those designated as higher-risk have at least 1 of these comorbidities. We distinguished patients as low-risk if they had no indication of any of these 27 comorbidities in their clinical records. Low-risk patients comprised 50.2% (n = 5 532 658/11 023 423) of urban and 50.9% (n = 264 435/519 953) of rural patients in this analysis.

### Main Outcome and Measures

The main outcome was SMM, defined by the US Centers for Disease Control and Prevention^2,16^ and its partners. Severe maternal morbidity includes unexpected outcomes of labor and delivery that result in significant short-term or long-term health consequences. We used *International Classification of Diseases, Clinical Modification* (ICD-CM) diagnosis and procedure codes to identify if any of the 20 indicators of SMM occurred during the delivery hospitalization. As is now recommended by the US Health Resources and Services Administration and others, we did not include blood products transfusion as an indicator of SMM.^8,9^ The SMM indicators represent either serious complications of pregnancy or delivery, such as sepsis or acute kidney failure, or procedures used to manage serious conditions, such as mechanical ventilation or hysterectomy.

Measured covariates included maternal age, primary payer at the time of childbirth, educational attainment, and maternal race and ethnicity, all of which are associated with SMM.^17,18,19^ All covariates are measured from the birth certificate records, except for primary payer, which come from patient discharge records for all states, and race and ethnicity, which come from the patient discharge records for the state of South Carolina.

### Statistical Analysis

First, we calculated the distribution of hospital birth volume and SMM for hospitals located in rural and urban US counties. Then, we measured the distribution of patient characteristics by hospital birth volume category (stratified by rural/urban). We estimated risk ratios and 95% CIs for the association between birth volume category and SMM using logistic regression models, with robust standard errors to account for hospital-level clustering. We included year fixed effects for all analyses. Analyses were stratified by rural or urban hospital location, because rural and urban hospitals had different birth volume distributions and distinct volume category definitions. We then stratified both the rural and urban analyses by clinical risk, modeling the association between volume category and risk of SMM for low-risk and higher-risk obstetric patients. Because SMM is a relatively rare outcome, we reported risk ratios, rather than odds ratios, as risk ratios have greater clinical interpretability than odds ratios and are similar when the outcome examined is rare.

A total of 11 543 376 records were included in the analysis (eFigure in Supplement 1). We included all linked data for obstetric patients aged 12 to 55 years. There were 460 records excluded based on age. This analysis also required that the data included county, and 16 850 records were excluded because rural/urban location could not be determined. Missing data for other variables were included as a “missing” category, and sensitivity analysis was conducted with complete cases only.

Obstetric patients who are transferred have elevated risk of SMM and may be transferred because of this risk.^20^ This analysis was based on the hospital where the childbirth occurred, and 0.13% (n = 15 483) of patients included in this analysis had hospital records indicating that they were transferred prior to childbirth, and 0.22% (n = 25 044) were transferred after childbirth.

We conducted a series of sensitivity analyses (eTables 1-5 in Supplement 1). First, blood transfusion codes were not included in this definition of SMM, consistent with current recommendations and owing to potential coding differences across hospitals.^21,22^ Second, we assessed the stability of results using alternate volume cut points. Specifically, we categorized hospitals as rural or urban and then divided hospitals into quartiles by birth volume and conducted the analysis with volume quartiles; results were substantively unchanged. Third, we conducted the main analyses stratifying by state. Fourth, we conducted a complete case analysis, excluding any records with missing data. Fifth, recognizing that obstetric transfer, while rare, may be more common for rural vs urban patients,^23^ we conducted an analysis excluding patients with indication of transfer. Results were robust to all sensitivity analyses. Analyses were conducted using Stata, version 17 (StataCorp LLC).

## Results

More than 11 million urban births and 519 953 rural births were included. The Figure shows a scatterplot of the association between hospital obstetric volume and SMM for rural and urban hospitals. Rural hospitals were concentrated at the lower end of obstetric volume, where there was also wide variation in SMM incidence. Table 1 shows the associations between birth volume category and SMM for hospitals in urban and rural counties, as well as unadjusted risk ratios and adjusted risk ratios (ARRs) across volume categories. Patient characteristics across volume categories are shown in eTable 6 (urban) and eTable 7 (rural) in Supplement 1. For both urban and rural hospitals, the referent category is the highest birth volume category. The rate of SMM varied from 0.73% to 0.50% across hospital volume categories (high to low) for urban hospitals. Unadjusted risk ratios indicated lower rates of SMM for lower volume urban hospitals, compared with higher volume urban hospitals (>2000 births per year). After adjusting for patient and clinical characteristics, there was no significant association between birth volume category and SMM for urban hospitals.

**Figure.**
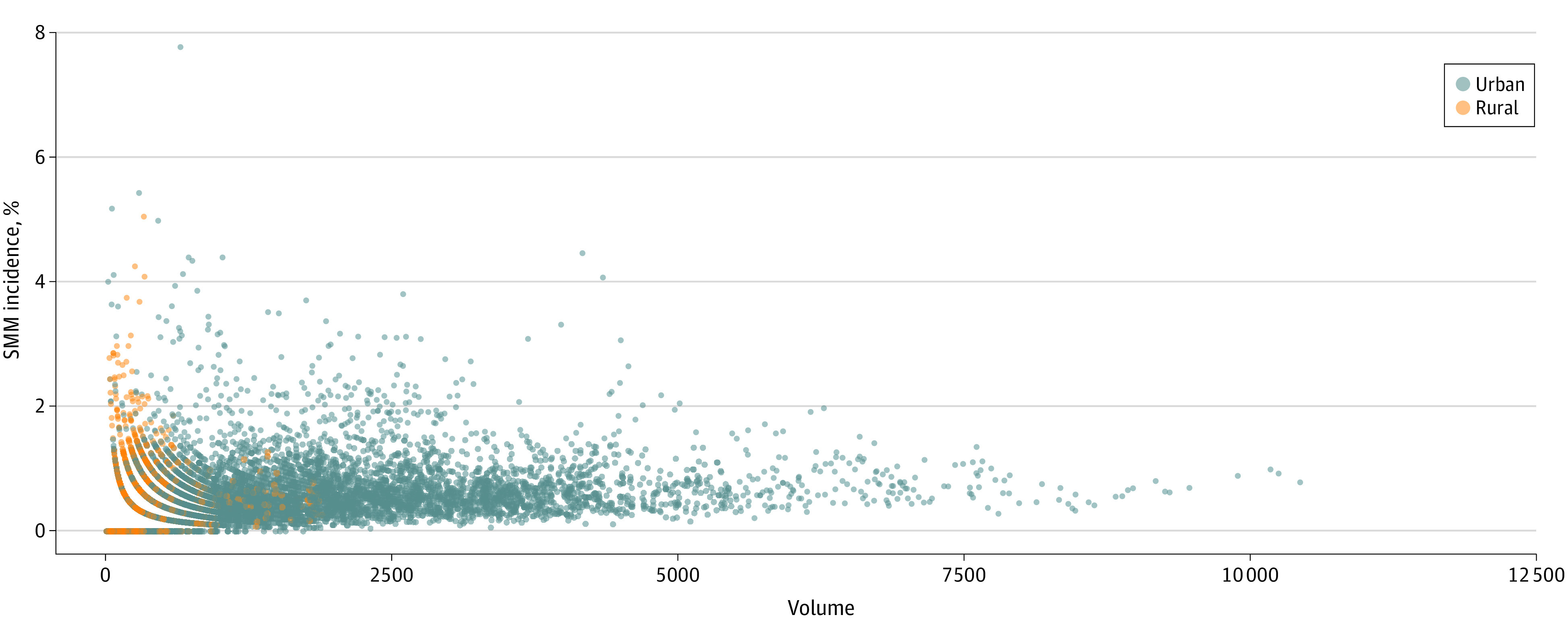
Associations Between Hospital Obstetric Volume and Severe Maternal Morbidity (SMM) Incidence for US Hospitals in Urban and Rural US Counties

**Table 1.** Associations Between Annual Birth Volume Category and Severe Maternal Morbidity (SMM) for Hospitals in Urban and Rural US Counties

Birth volume category	Total patients, No.	SMM incidence, No. (%)	Risk ratio (95% CI)	
			Unadjusted	Adjusted
**Urban counties**				
Low (10-500 births)	261 553	1316 (0.50)	0.69 (0.61-0.79)	1.00 (0.90-1.11)
Medium (501-1000 births)	860 892	4908 (0.57)	0.78 (0.66-0.93)	1.01 (0.90-1.13)
Medium-high (1001-2000 births)	2 535 466	16 476 (0.65)	0.89 (0.80-1.00)	1.03 (0.96-1.10)
High (>2000 births)	7 365 512	53 507 (0.73)	1 [Reference]	1 [Reference]
**Rural counties**				
Low (10-110 births)	8182	57 (0.70)	1.48 (1.01-2.18)	1.65 (1.14-2.39)
Medium (111-240 births)	59 374	324 (0.55)	1.16 (0.90-1.49)	1.37 (1.10-1.70)
Medium-high (241-460 births)	175 176	967 (0.55)	1.17 (0.96-1.44)	1.26 (1.05-1.51)
High (>460 births)	277 221	1304 (0.47)	1 [Reference]	1 [Reference]

Among rural hospitals, the SMM rate varied from 0.47% to 0.70% across hospital volume categories (high to low). Unadjusted risk ratios indicated higher SMM rates among lower-volume rural hospitals, compared with higher rural hospitals with greater than 460 births per year, but the difference was only statistically significant between the lowest volume category and the highest. However, after adjusting for relevant patient and clinical characteristics, the risk of SMM was elevated for patients who gave birth at rural hospitals with 10 to 110 annual births (ARR, 1.65, 95% CI, 1.14-2.39), 111 to 240 annual births (ARR, 1.37; 95% CI, 1.10-1.70), and 241 to 460 annual births (ARR, 1.26; 95% CI, 1.05-1.51), compared with those who gave birth at rural hospitals with greater than 460 births annually.

There was a wide range of facilities within the low birth volume category of urban hospitals (10-500 births). To assess SMM rates at rural and urban hospitals of similar birth volume, Table 2 shows the association between SMM and birth volume for low-volume urban hospitals, using the same cut points described above for rural hospital volume categories; there was no significant association detected between obstetric volume and SMM among low-volume urban hospitals.

**Table 2.** Associations Between Annual Birth Volume Category and Severe Maternal Morbidity (SMM) for Hospitals in Urban and Rural US Counties, Only Including Urban Hospitals With 600 or Fewer Annual Births

Births, No.	Total patients, No.	SMM incidence, No. (%)	Risk ratio (95% CI)	
			Unadjusted	Adjusted
**Low-volume hospitals in urban counties**				
10-110	5308	37 (0.70)	1.24 (0.88-1.76)	1.28 (0.93-1.77)
111-240	24 238	98 (0.40)	0.72 (0.54-0.95)	0.90 (0.72-1.12)
241-460	178 076	875 (0.49)	0.87 (0.71-1.08)	0.96 (0.83-1.12)
461-600	176 027	989 (0.56)	1 [Reference]	1 [Reference]
**Rural counties**				
10-110	8182	57 (0.70)	1.48 (1.01-2.18)	1.65 (1.14-2.39)
111-240	59 374	324 (0.55)	1.16 (0.90-1.49)	1.37 (1.10-1.70)
241-460	175 176	967 (0.55)	1.17 (0.96-1.44)	1.26 (1.05-1.51)
>460	277 221	1304 (0.47)	1 [Reference]	1 [Reference]

Table 3 shows the association between hospital birth volume category and SMM for low-risk and higher-risk obstetric patients at urban hospitals. There was no statistically significant association between birth volume category and SMM, for either low-risk or higher-risk patients who gave birth at urban hospitals, after adjusting for patient and clinical characteristics.

**Table 3.** Association Between Birth Volume Category and Severe Maternal Morbidity for Higher-risk and Low-risk Obstetric Patients at Hospitals in Urban Counties

Annual birth volume	Risk ratio (95% CI)			
	Higher-risk patients		Low-risk patients	
	Unadjusted	Adjusted	Unadjusted	Adjusted
Low (10-500 births)	0.71 (0.63-0.80)	1.04 (0.93-1.15)	0.84 (0.69-1.03)	0.92 (0.76-1.11)
Medium (501-1000 births)	0.81 (0.69-0.96)	1.06 (0.94-1.19)	0.80 (0.67-0.94)	0.85 (0.73-1.00)
Medium-high (1001-2000 births)	0.91 (0.82-1.00)	1.04 (0.98-1.11)	0.95 (0.83-1.09)	0.99 (0.87-1.14)
High (>2000 births)	1 [Reference]	1 [Reference]	1 [Reference]	1 [Reference]

Table 4 shows the association between hospital birth volume category and SMM for low-risk and higher-risk obstetric patients at rural hospitals. After adjusting for clinical and patient characteristics, the risk ratios for both low-risk and higher-risk patients were elevated at lower-volume facilities, compared with rural hospitals with more than 460 births a year. Among higher-risk patients (those with 1 or more comorbidities that put them at greater risk for SMM), the risk ratio was elevated for low (ARR, 1.49; 95% CI, 1.01-2.20), medium (ARR, 1.30; 95% CI, 1.03-1.65), and medium-high (ARR, 1.16; 95% CI, 0.95-1.43) volume hospitals, compared with high-volume rural hospitals, though the difference between medium-high and high volume rural hospitals was not statistically significant for higher-risk patients. Associations between birth volume and SMM were more pronounced for low-risk patients at rural hospitals (those with no comorbidities). For these patients, the risk of SMM more than doubled for patients giving birth at the lowest volume hospitals, compared with the highest volume category (ARR, 2.32; 95% CI, 1.32-4.07), and rates were also elevated for rural patients at medium (ARR, 1.66; 95% CI, 1.20-2.28) and medium-high (ARR, 1.68; 95% CI, 1.29-2.18) volume hospitals, compared with high birth volume hospitals in rural counties.

**Table 4.** Association Between Birth Volume Category and Severe Maternal Morbidity for Higher-risk and Low-risk Obstetric Patients at Hospitals in Rural Counties

Annual birth volume	Risk ratio (95% CI)			
	Higher-risk patients		Low-risk patients	
	Unadjusted	Adjusted	Unadjusted	Adjusted
Low (10-110 births)	1.29 (0.87-1.90)	1.49 (1.01-2.20)	2.37 (1.31-4.30)	2.32 (1.32-4.07)
Medium (111-240 births)	1.09 (0.84-1.41)	1.30 (1.03-1.65)	1.60 (1.15-2.22)	1.66 (1.20-2.28)
Medium-high (241-460 births)	1.05 (0.85-1.29)	1.16 (0.95-1.43)	1.54 (1.13-2.10)	1.68 (1.29-2.18)
High (>460 births)	1 [Reference]	1 [Reference]	1 [Reference]	1 [Reference]

## Discussion

For hospitals located in rural counties, risk of SMM was elevated for patients who gave birth at lower volume hospitals, compared with patients who delivered at higher volume hospitals. Increased risk of SMM occurred for both low-risk and higher-risk obstetric patients who delivered at rural hospitals with lower birth volumes, with low-risk patients who gave birth at low-volume rural facilities (10-110 births annually) having more than double the risk of SMM, compared with low-risk patients who gave birth at high–birth volume rural hospitals (>460 births annually).

Notably, the patterns of SMM rates across volume categories were distinct for hospitals in rural and urban counties. In urban counties, the highest unadjusted rates of SMM were seen at the highest volume hospitals, whereas in rural counties, higher rates of SMM were in the lowest birth volume hospitals. The differences between unadjusted risk ratios and ARRs, where risk differences across volume categories were attenuated, generally implied effective referral patterns of the sicker, higher-risk patients to hospitals with appropriate services for these patients in urban counties. That is, before risk adjustment, patients at hospitals with lower volume in urban areas had lower risk of SMM; this was particularly notable among higher-risk patients. This association was no longer significant after risk adjustment, which implies that in urban areas, lower-volume hospitals likely referred clinically complex patients to the high-volume hospitals, which generally have more resources to care for high-acuity patients.^24,25,26^

However, this pattern did not hold for patients in rural areas, especially for low-risk patients. Our finding of elevated rates of SMM among lower-volume rural hospitals is consistent with prior literature,^27^ and this study adds to what is known by further distinguishing low-risk and higher-risk patients. That the association between birth volume and SMM was amplified among low-risk patients in rural counties is concerning for efforts to support local access to childbirth care for low-risk patients in rural communities. In several international contexts, national (Portugal) or state/provincial (British Columbia, Canada) governments have successfully implemented large-scale regionalization policies that closed small-volume rural obstetric units in order to care for obstetric patients in higher-volume settings.^10,28^ However, closure of low-volume obstetric units is not recommended as a policy strategy in the US based on the current findings. In urban counties, there was no significant SMM decrement detected for hospitals with birth volume less than 500 compared with greater than 2000 births per year. In rural counties, closure of hospital-based obstetric units, which are at greater risk of closure when they are lower volume and located in more remote areas, is associated with increases in emergency birth and preterm birth,^29^ and travel distances are associated with adverse infant and maternal outcomes.^30^

Rather than implying a policy strategy of consolidation and closure, these findings and the available evidence suggest a need for tailored quality improvement resources for rural hospitals, greater investment in rural clinician training, and establishment of referral or transfer networks for rural hospitals to improve obstetric patient safety.^31^ Participation in state perinatal care quality collaboratives may hold promise for rural hospitals,^32^ but many maternal and perinatal quality improvement initiatives are not rural-relevant or rural-tailored. For example, currently available obstetric care quality improvement initiatives have had tremendous success in reducing SMM, but the cost-effectiveness of adopting care bundles decreases as birth volume decreases,^33^ requiring attention to the financial feasibility in low-volume settings, especially in rural areas.^6^

To address the elevated risks of SMM for both low-risk and higher-risk patients who give birth at low-volume hospitals in rural counties, investments and adjustments to current policies and programs may help. Medicaid finances more than half of births at rural hospitals, and low-volume payment enhancements could address resource constraints, clinician availability and training, and financial viability concerns faced by small-volume rural hospitals.^6^ Additionally, the Centers for Medicare & Medicaid Services (CMS) recently launched a plan to establish a Birthing-Friendly Hospital designation.^34^ Such a designation holds potential to improve obstetric services in rural settings if it incorporates rural-specific resources and investments to help low-volume rural hospitals achieve and maintain evidence-based support services and an adequate workforce, as well as simulation and other training to maintain staff skills.^6,35^ Finally, the recent CMS Rural Emergency Hospital designation will support rural hospitals in converting from an inpatient facility to an emergency-focused facility with the capacity to stabilize and transfer patients. Most hospitals likely to qualify for this designation are smaller-volume rural facilities,^36^ and investments in resources for emergency obstetrics and appropriate transfer capacity for obstetric patients at Rural Emergency Hospitals may improve safety for people giving birth in rural counties.

In addition to investments in critical access obstetrics in rural communities and to clinical care and support, policy attention to the broader social determinants of maternal and infant health in remote, rural communities is important.^37,38^ In particular, attention to the intersection of structural racism and structural urbanism requires investment in rural communities with substantial Black and Indigenous populations, where maternal health risks are most acute and access to obstetric care is most limited.^17,39,40,41,42^

### Limitations

Measuring rurality is complex, and using county-based measures has drawbacks, especially given the variability in the geographic size of a county and the distribution of people, infrastructure, and resources across and within counties.^3^ Such measures may classify remote hospitals in geographically large counties that contain urban centers as “urban.” Additionally, rurality is a continuum, not a dichotomy, but is measured dichotomously in this analysis. Second, the linked vital statistic–hospital discharge administrative data used in this analysis do not contain vital signs, laboratory values, clinical notes, obstetric care workforce, or details on social determinants of SMM, but provide advantages beyond using birth certificates or discharge data alone. Third, unmeasured patient risk factors could potentially bias results downward (toward a lack of association between birth volume and SMM) if there are unmeasured patient complications that disproportionately occur at higher-volume hospitals. Fourth, these data come from 4 geographically and demographically diverse US states, representing more than a quarter of all US births, but some measured characteristics differ across states (eTable 8 in Supplement 1), 2 states did not have data available for all study years, and there is limited representation from highly rural states, which may have distinct contexts for access, quality, and safety of obstetric care.^4,5^ Fifth, these data do not contain comprehensive information on patient referral or transfer, limiting our ability to understand their role in the associations uncovered in this analysis.

## Conclusions

In this cross-sectional study of births in US rural and urban counties, risk of SMM was elevated for both low-risk and higher-risk obstetric patients who gave birth in lower-volume hospitals in rural counties, compared with similar patients who gave birth at rural hospitals with higher annual birth volume. These findings imply a need for tailored quality improvement strategies for lower-volume hospitals in rural communities.

## Notes

Supplement 1. **eFigure:** Study population flowchart **eTable 1:** Associations between annual birth volume category and SMM (with transfusion) for hospitals in urban and rural U.S. counties **eTable 2:** Associations between annual birth volume category and SMM for hospitals in urban and rural U.S. counties, alternate birth volume categories **eTable 3:** Associations between annual birth volume category and SMM for hospitals with in urban and rural U.S. counties, stratified by state **eTable 4:** Associations between annual birth volume category and SMM for hospitals in urban and rural U.S. counties, complete case analysis (excluding missing data) **eTable 5:** Associations between annual birth volume category and SMM for hospitals in urban and rural U.S. counties, excluding patients with transfer indications **eTable 6:** Characteristics of obstetric patients at hospitals in urban US counties, by birth volume category **eTable 7:** Characteristics of obstetric patients at hospitals in rural US counties, by birth volume category **eTable 8:** Characteristics of obstetric patients in the study population, based on state of residence

Supplement 2.
Data Sharing Statement
