# Supplementary material for: Obstetric Volume and Severe Maternal Morbidity Among Low-Risk and Higher-Risk Patients Giving Birth at Rural and Urban US Hospitals
Source: JAMA Health Forum. 2023 Jun 24;4(6):e232110. doi: 10.1001/jamahealthforum.2023.2110 (PMC10290751; doi:10.1001/jamahealthforum.2023.2110)
Supplement: Supplement 1. — eFigure: Study population flowchart eTable 1: Associations between annual birth volume category and SMM (with transfusion) for hospitals in urban and rural U.S. counties eTable 2: Associations between annual birth volume category and SMM for hospitals in urban and rural U.S. counties, alternate birth volume categories eTable 3: Associations between annual birth volume category and SMM for hospitals with in urban and rural U.S. counties, stratified by state eTable 4: Associations between annual birth volume category and SMM for hospitals in urban and rural U.S. counties, complete case analysis (excluding missing data) eTable 5: Associations between annual birth volume category and SMM for hospitals in urban and rural U.S. counties, excluding patients with transfer indications eTable 6: Characteristics of obstetric patients at hospitals in urban US counties, by birth volume category eTable 7: Characteristics of obstetric patients at hospitals in rural US counties, by birth volume category eTable 8: Characteristics of obstetric patients in the study population, based on state of residence [file jamahealthforum-e232110-s001.pdf]

Supplemental Online Content

Kozhimannil KB, Leonard SA, Handley SC, et al. Obstetric volume and severe maternal morbidity among low-risk and higher-risk patients giving birth at rural and urban US hospitals. *JAMA Health Forum*. 2023;4(6):e232110. doi:10.1001/jamahealthforum.2023.2110

eFigure: Study population flowchart

eTable 1: Associations between annual birth volume category and SMM (with transfusion) for hospitals in urban and rural U.S. counties

eTable 2: Associations between annual birth volume category and SMM for hospitals in urban and rural U.S. counties, alternate birth volume categories

eTable 3: Associations between annual birth volume category and SMM for hospitals with in urban and rural U.S. counties, stratified by state

eTable 4: Associations between annual birth volume category and SMM for hospitals in urban and rural U.S. counties, complete case analysis (excluding missing data)

eTable 5: Associations between annual birth volume category and SMM for hospitals in urban and rural U.S. counties, excluding patients with transfer indications

eTable 6: Characteristics of obstetric patients at hospitals in urban US counties, by birth volume category

eTable 7: Characteristics of obstetric patients at hospitals in rural US counties, by birth volume category

eTable 8: Characteristics of obstetric patients in the study population, based on state of residence

This supplemental material has been provided by the authors to give readers additional information about their work.

© 2023 Kozhimannil KB et al. *JAMA Health Forum.*

eFigure: Study population flowchart

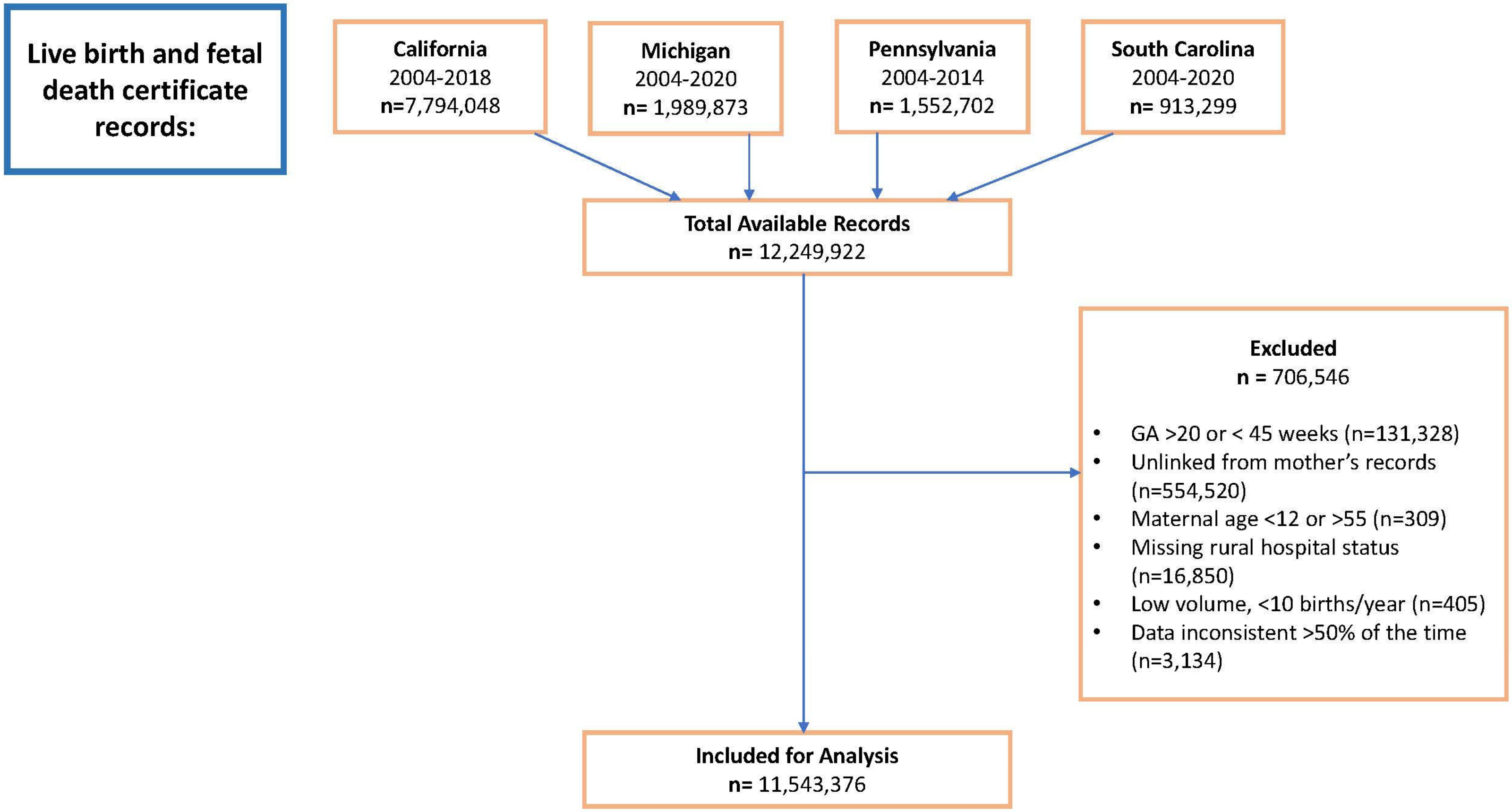

© 2023 Kozhimannil KB et al. *JAMA Health Forum.*

eTable 1: Associations between annual birth volume category and SMM (with transfusion) for hospitals in urban and rural U.S. counties

	SMM Incidence n (%)	Unadjusted RR (95% CI)	Adjusted RR (95% CI)
**Urban Counties**			
Low (10-500 births)	3637 (1.39%)	0.89 (0.75-1.04)	1.3 (1.12-1.5)
Medium (501-1000 births)	11846 (1.38%)	0.87 (0.76-1.01)	1.12 (1.02-1.24)
Medium-high (1001-2000 births)	37170 (1.47%)	0.93 (0.84-1.03)	1.09 (1-1.17)
High (>2000 births)	115473 (1.57%)	REF	REF
**Rural Counties**			
Low (10-110 births)	159 (1.94%)	1.59 (1.03-2.47)	1.79 (1.24-2.6)
Medium (111-240 births)	849 (1.43%)	1.16 (0.91-1.49)	1.34 (1.07-1.67)
Medium-high (241-460 births)	2728 (1.56%)	1.27 (1.02-1.58)	1.25 (0.99-1.59)
High (>460 births)	3419 (1.23%)	REF	REF

© 2023 Kozhimannil KB et al. *JAMA Health Forum.*

eTable 2: Associations between annual birth volume category and SMM for hospitals in urban and rural U.S. counties, alternate birth volume categories

	Severe Maternal Morbidity Rate	Unadjusted RR (95% CI)	Adjusted RR (95% CI)
**Urban**			
Q1-Low: 11-767	3692 (0.55%)	0.77 90.66-0.89)	1.05 (0.95-1.16)
Q2-Medium: 768-1521	9774 (0.6%)	0.83 (0.73-0.95)	1.04 (0.95-1.14)
Q3-Medium-High: 1521-2602	21105 (0.72%)	1.00 (0.89-1.12)	1.09 (1.00-1.18)
Q4-High: > 2602	41636 (0.72%)	REF	REF
**Rural**			
Q1-Low: 11-207	243 (0.53%)	1.14 (0.90-1.43)	1.34 (1.11-1.63)
Q2-Medium: 208-320	529 (0.6%)	1.27 (1.04-1.55)	1.35 (1.12-1.62)
Q3-Medium-High: 321-486	682 (0.52%)	1.10 (0.88-1.39)	1.22 (0.99-1.52)
Q4-High: > 486	1198 (0.47%)	REF	REF

© 2023 Kozhimannil KB et al. *JAMA Health Forum.*

eTable 3: Associations between annual birth volume category and SMM for hospitals with in urban and rural U.S. counties, stratified by state

		Severe Maternal Morbidity Rate	Unadjusted RR (95% CI)	Adjusted RR (95% CI)
CA	**Urban**			
	Volume category 1	621 (0.54%)	0.80 (0.67-0.96)	1.01 (0.87-1.17)
	Volume category 2	2205 (0.53%)	0.79 (0.61-1.02)	0.91 (0.77-1.07)
	Volume category 3	9670 (0.65%)	0.96 (0.83-1.12)	1.02 (0.93-1.12)
	Volume category 4	35577 (0.68%)	REF	REF
	**Rural**			
	Volume category 1	22 (0.89%)	2.03 (1.04-3.94)	2.17 (1.30-3.64)
	Volume category 2	101 (0.57%)	1.29 (0.80-2.10)	1.37 (0.90-2.09)
	Volume category 3	180 (0.61%)	1.38 (1.00-1.92)	1.35 (0.98-1.89)
	Volume category 4	225 (0.44%)	REF	REF
MI	**Urban**			
	Volume category 1	166 (0.39%)	0.45 (0.33-0.60)	0.91 (0.74-1.13)
	Volume category 2	1283 (0.65%)	0.75 (0.51-1.10)	1.18 (0.89-1.57)
	Volume category 3	2884 (0.66%)	0.76 (0.59-0.97)	1.10 (0.96-1.27)
	Volume category 4	8937 (0.87%)	REF	REF
	**Rural**			
	Volume category 1	21 (0.64%)	1.35 (0.76-2.37)	1.64 (0.93-2.88)
	Volume category 2	107 (0.49%)	1.03 (0.69-1.54)	1.39 (0.98-1.98)
	Volume category 3	450 (0.51%)	1.07 (0.74-1.55)	1.24 (0.85-1.82)
	Volume category 4	490 (0.48%)	REF	REF

© 2023 Kozhimannil KB et al. *JAMA Health Forum.*

PA	**Urban**			
	Volume category 1	316 (0.46%)	0.58 (0.45-0.76)	0.93 (0.71-1.22)
	Volume category 2	881 (0.53%)	0.68 (0.54-0.85)	1.05 (0.85-1.29)
	Volume category 3	2548 (0.65%)	0.83 (0.66-1.04)	1.03 (0.88-1.20)
	Volume category 4	5123 (0.78%)	REF	REF
	**Rural**			
	Volume category 1	7 (0.65%)	1.68 (0.75-3.78)	1.75 (0.84-3.61)
	Volume category 2	85 (0.55%)	1.41 (0.85-2.32)	1.46 (0.91-2.36)
	Volume category 3	172 (0.53%)	1.35 (1.03-1.76)	1.50 (1.12-2.02)
	Volume category 4	234 (0.39%)	REF	REF
SC	**Urban**			
	Volume category 1	213 (0.6%)	0.66 (0.43-0.99)	1.11 (0.84-1.47)
	Volume category 2	539 (0.64%)	0.70 (0.47-1.05)	1.08 (0.83-1.40)
	Volume category 3	1374 (0.62%)	0.68 (0.48-0.97)	0.93 (0.76-1.13)
	Volume category 4	3870 (0.91%)	REF	REF
	**Rural**			
	Volume category 1	7 (0.52%)	0.92 (0.33-2.57)	0.99 (0.37-2.65)
	Volume category 2	31 (0.73%)	1.30 (0.85-2.00)	1.27 (0.86-1.88)
	Volume category 3	165 (0.68%)	1.21 (0.94-1.56)	1.12 (0.94-1.33)
	Volume category 4	355 (0.56%)	REF	REF

© 2023 Kozhimannil KB et al. *JAMA Health Forum.*

eTable 4: Associations between annual birth volume category and SMM for hospitals in urban and rural U.S. counties, complete case analysis (excluding missing data)

	SMM Incidence n (%)	Unadjusted RR (95% CI)	Adjusted RR (95% CI)
**Urban Counties**			
Low (10-500 births)	1257 (0.49%)	0.68 (0.60-0.77)	0.98 (0.88-1.10)
Medium (501-1000 births)	4755 (0.56%)	0.78 (0.66-0.93)	1.00 (0.89-1.13)
Medium-high (1001-2000 births)	15808 (0.64%)	0.89 (0.80-1.00)	1.02 (0.96-1.10)
High (>2000 births)	50944 (0.72%)	REF	REF
**Rural Counties**			
Low (10-110 births)	52 (0.66%)	1.43 (0.96-2.12)	1.63 (1.13-2.35)
Medium (111-240 births)	311 (0.53%)	1.15 (0.89-1.49)	1.33 (1.06-1.68)
Medium-high (241-460 births)	931 (0.54%)	1.16 (0.95-1.43)	1.24 (1.03-1.50)
High (>460 births)	274316 (0.46%)	REF	REF

© 2023 Kozhimannil KB et al. *JAMA Health Forum.*

eTable 5: Associations between annual birth volume category and SMM for hospitals in urban and rural U.S. counties, excluding patients with transfer indications

	SMM Incidence n (%)	Unadjusted RR (95% CI)	Adjusted RR (95% CI)
**Urban Counties**			
Low (10-500 births)	1241 (0.48%)	0.67 (0.59-0.77)	0.97 (0.87-1.08)
Medium (501-1000 births)	4726 (0.55%)	0.78 (0.66-0.92)	1.00 (0.89-1.12)
Medium-high (1001-2000 births)	15849 (0.63%)	0.89 (0.80-0.99)	1.02 (0.95-1.09)
High (>2000 births)	51630 (0.70%)	REF	REF
**Rural Counties**			
Low (10-110 births)	54 (0.66%)	1.47 (1.01-2.15)	1.65 (1.14-2.38)
Medium (111-240 births)	311 (0.52%)	1.16 (0.90-1.50)	1.36 (1.09-1.69)
Medium-high (241-460 births)	917 (0.52%)	1.16 (0.94-1.44)	1.24 (1.02-1.50)
High (>460 births)	1248 (0.45%)	REF	REF

© 2023 Kozhimannil KB et al. *JAMA Health Forum.*

eTable 6: Characteristics of obstetric patients at hospitals in urban US counties, by birth volume category

	Total	Low	Medium	Medium-High	High	p-value
	N=11,023,423	N=261,553	N=860,892	N=2,535,466	N=7,365,512	
Maternal Age at Admission (years)	28.48 (6.15)	27.17 (6.01)	27.83 (6.03)	28.33 (6.11)	28.66 (6.17)	<0.001
Maternal Age Category						<0.001
<25	3,138,417 (28.47%)	96,269 (36.81%)	276,574 (32.13%)	741,445 (29.24%)	2,024,129 (27.48%)	
25-34	5,933,544 (53.83%)	131,758 (50.38%)	456,750 (53.06%)	1,363,857 (53.79%)	3,981,179 (54.05%)	
>=35	1,951,462 (17.70%)	33,526 (12.82%)	127,568 (14.82%)	430,164 (16.97%)	1,360,204 (18.47%)	
Maternal Race/Ethnicity						<0.001
AI/AN	25,897 (0.23%)	897 (0.34%)	1,863 (0.22%)	6,482 (0.26%)	16,655 (0.23%)	
Asian	1,108,997 (10.06%)	9,677 (3.70%)	49,995 (5.81%)	206,924 (8.16%)	842,401 (11.44%)	
Black	1,169,224 (10.61%)	20,657 (7.90%)	86,857 (10.09%)	284,881 (11.24%)	776,829 (10.55%)	
Hispanic	3,984,383 (36.14%)	69,570 (26.60%)	250,068 (29.05%)	827,003 (32.62%)	2,837,742 (38.53%)	
Pacific Islander	31,566 (0.29%)	418 (0.16%)	1,869 (0.22%)	7,423 (0.29%)	21,856 (0.30%)	
White	4,422,185 (40.12%)	153,257 (58.60%)	447,102 (51.93%)	1,135,736 (44.79%)	2,686,090 (36.47%)	
Other/Multiple	269,157 (2.44%)	6,805 (2.60%)	22,227 (2.58%)	62,553 (2.47%)	177,572 (2.41%)	
Missing	12,014 (0.11%)	272 (0.10%)	911 (0.11%)	4,464 (0.18%)	6,367 (0.09%)	
Maternal Education Category						<0.001
Missing	307,542 (2.79%)	3,867 (1.48%)	11,739 (1.36%)	54,937 (2.17%)	236,999 (3.22%)	
No HS	601,144 (5.45%)	13,733 (5.25%)	45,962 (5.34%)	136,403 (5.38%)	405,046 (5.50%)	
Some HS	1,524,889 (13.83%)	45,528 (17.41%)	127,519 (14.81%)	350,831 (13.84%)	1,001,011 (13.59%)	
HS Degree	2,744,173 (24.89%)	81,813 (31.28%)	245,985 (28.57%)	651,751 (25.71%)	1,764,624 (23.96%)	
Some College	2,781,434 (25.23%)	70,044 (26.78%)	238,755 (27.73%)	656,361 (25.89%)	1,816,274 (24.66%)	
4 Yr College	1,928,154 (17.49%)	32,386 (12.38%)	128,815 (14.96%)	439,626 (17.34%)	1,327,327 (18.02%)	
>4 Yrs College	1,136,087 (10.31%)	14,182 (5.42%)	62,117 (7.22%)	245,557 (9.68%)	814,231 (11.05%)	
Maternal Payor Category						<0.001
Missing	1,283 (0.01%)	68 (0.03%)	97 (0.01%)	627 (0.02%)	491 (0.01%)	
Private	5,581,410 (50.63%)	106,618 (40.76%)	390,035 (45.31%)	1,251,135 (49.35%)	3,833,622 (52.05%)	

© 2023 Kozhimannil KB et al. *JAMA Health Forum.*

Government	5,090,005 (46.17%)	145,588 (55.66%)	439,435 (51.04%)	1,188,385 (46.87%)	3,316,597 (45.03%)	
Self Pay	239,456 (2.17%)	6,817 (2.61%)	20,328 (2.36%)	59,335 (2.34%)	152,976 (2.08%)	
Other	111,269 (1.01%)	2,462 (0.94%)	10,997 (1.28%)	35,984 (1.42%)	61,826 (0.84%)	
Comorbidity Count	0.81 (1.06)	0.69 (0.93)	0.75 (0.99)	0.78 (1.03)	0.84 (1.09)	<0.001
Comorbidity score for SMM	3.87 (6.36)	3.05 (5.17)	3.36 (5.62)	3.62 (6.02)	4.04 (6.58)	<0.001
Comorbidity score for non-transfusion SMM	4.81 (8.57)	3.65 (6.79)	4.04 (7.44)	4.48 (8.08)	5.05 (8.90)	<0.001
High Risk: Comorbidity Count >=1						<0.001
Low Risk	5,532,658 (50.19%)	140,526 (53.73%)	447,791 (52.01%)	1,297,779 (51.19%)	3,646,562 (49.51%)	
1 or more comorbidities	5,490,765 (49.81%)	121,027 (46.27%)	413,101 (47.99%)	1,237,687 (48.81%)	3,718,950 (50.49%)	

© 2023 Kozhimannil KB et al. *JAMA Health Forum.*

eTable 7: Characteristics of obstetric patients at hospitals in rural US counties, by birth volume category

	Total	Low	Medium	Medium-High	High	p-value
	N=519,953	N=8,182	N=59,374	N=175,176	N=277,221	
Maternal Age at Admission (years)	26.55 (5.74)	26.72 (5.86)	26.24 (5.76)	26.36 (5.69)	26.74 (5.75)	<0.001
Maternal Age Category						<0.001
<25	209,125 (40.22%)	3,226 (39.43%)	25,441 (42.85%)	73,040 (41.70%)	107,418 (38.75%)	
25-34	258,507 (49.72%)	4,038 (49.35%)	28,248 (47.58%)	85,533 (48.83%)	140,688 (50.75%)	
>=35	52,321 (10.06%)	918 (11.22%)	5,685 (9.57%)	16,603 (9.48%)	29,115 (10.50%)	
Maternal Race/Ethnicity						<0.001
AI/AN	10,988 (2.11%)	119 (1.45%)	1,416 (2.38%)	4,326 (2.47%)	5,127 (1.85%)	
Asian	4,612 (0.89%)	63 (0.77%)	469 (0.79%)	1,563 (0.89%)	2,517 (0.91%)	
Black	46,585 (8.96%)	793 (9.69%)	2,857 (4.81%)	13,634 (7.78%)	29,301 (10.57%)	
Hispanic	40,766 (7.84%)	990 (12.10%)	6,970 (11.74%)	12,229 (6.98%)	20,577 (7.42%)	
Pacific Islander	296 (0.06%)	4 (0.05%)	52 (0.09%)	101 (0.06%)	139 (0.05%)	
White	401,294 (77.18%)	5,937 (72.56%)	45,905 (77.31%)	138,285 (78.94%)	211,167 (76.17%)	
Other/Multiple	14,628 (2.81%)	127 (1.55%)	1,640 (2.76%)	4,928 (2.81%)	7,933 (2.86%)	
Missing	784 (0.15%)	149 (1.82%)	65 (0.11%)	110 (0.06%)	460 (0.17%)	
Maternal Education Category						<0.001
Missing	4,398 (0.85%)	107 (1.31%)	499 (0.84%)	1,257 (0.72%)	2,535 (0.91%)	
No HS	14,395 (2.77%)	365 (4.46%)	1,911 (3.22%)	5,053 (2.88%)	7,066 (2.55%)	
Some HS	75,432 (14.51%)	1,267 (15.49%)	9,991 (16.83%)	26,640 (15.21%)	37,534 (13.54%)	
HS Degree	174,442 (33.55%)	2,961 (36.19%)	21,801 (36.72%)	63,192 (36.07%)	86,488 (31.20%)	
Some College	162,083 (31.17%)	2,302 (28.13%)	17,288 (29.12%)	52,859 (30.17%)	89,634 (32.33%)	
4 Yr College	61,945 (11.91%)	858 (10.49%)	5,463 (9.20%)	18,783 (10.72%)	36,841 (13.29%)	
>4 Yrs College	27,258 (5.24%)	322 (3.94%)	2,421 (4.08%)	7,392 (4.22%)	17,123 (6.18%)	
Maternal Payor Category						<0.001
Missing	62 (0.01%)	0 (0.00%)	8 (0.01%)	6 (0.00%)	48 (0.02%)	
Private	216,856 (41.71%)	2,948 (36.03%)	21,841 (36.79%)	69,157 (39.48%)	122,910 (44.34%)	
Government	281,874 (54.21%)	4,822 (58.93%)	35,521 (59.83%)	98,929 (56.47%)	142,602 (51.44%)	

© 2023 Kozhimannil KB et al. *JAMA Health Forum.*

Self Pay	8,185 (1.57%)	160 (1.96%)	987 (1.66%)	2,820 (1.61%)	4,218 (1.52%)	
Other	12,976 (2.50%)	252 (3.08%)	1,017 (1.71%)	4,264 (2.43%)	7,443 (2.68%)	
Comorbidity Count	0.77 (0.99)	0.73 (0.92)	0.72 (0.94)	0.81 (1.00)	0.75 (0.99)	<0.001
Comorbidity score for SMM	3.40 (5.47)	3.12 (4.99)	3.12 (5.09)	3.66 (5.55)	3.30 (5.50)	<0.001
Comorbidity score for non-transfusion SMM	4.07 (7.23)	3.75 (6.58)	3.65 (6.57)	4.25 (7.14)	4.06 (7.42)	<0.001
High Risk: Comorbidity Count >=1						<0.001
Low Risk	264,435 (50.86%)	4,138 (50.57%)	31,158 (52.48%)	84,821 (48.42%)	144,318 (52.06%)	
1 or more comorbidities	255,518 (49.14%)	4,044 (49.43%)	28,216 (47.52%)	90,355 (51.58%)	132,903 (47.94%)	

© 2023 Kozhimannil KB et al. *JAMA Health Forum.*

eTable 8: Characteristics of obstetric patients in the study population, based on state of residence

	Total	California	Michigan	Pennsylvania	South Carolina	p-value
	N=11,543,376	N=7,368,876	N=1,924,318	N=1,393,587	N=856,595	
Maternal Age at Admission (years)	28.39 (6.14)	28.76 (6.23)	27.86 (5.84)	28.08 (5.99)	26.98 (5.92)	<0.001
Maternal Age Category						<0.001
<25	3,347,542 (29.00%)	2,015,856 (27.36%)	589,911 (30.66%)	419,741 (30.12%)	322,034 (37.59%)	
25-34	6,192,051 (53.64%)	3,925,659 (53.27%)	1,071,192 (55.67%)	760,979 (54.61%)	434,221 (50.69%)	
>=35	2,003,783 (17.36%)	1,427,361 (19.37%)	263,215 (13.68%)	212,867 (15.27%)	100,340 (11.71%)	
Maternal Race/Ethnicity						<0.001
AI/AN	36,885 (0.32%)	25,302 (0.34%)	8,152 (0.42%)	737 (0.05%)	2,694 (0.31%)	
Asian	1,113,609 (9.65%)	992,225 (13.47%)	67,730 (3.52%)	43,538 (3.12%)	10,116 (1.18%)	
Black	1,215,809 (10.53%)	387,974 (5.27%)	356,842 (18.54%)	193,587 (13.89%)	277,406 (32.38%)	
Hispanic	4,025,149 (34.87%)	3,742,844 (50.79%)	131,708 (6.84%)	110,290 (7.91%)	40,307 (4.71%)	
Pacific Islander	31,862 (0.28%)	30,980 (0.42%)	500 (0.03%)	382 (0.03%)	0 (0.00%)	
White	4,823,479 (41.79%)	2,031,127 (27.56%)	1,316,755 (68.43%)	997,680 (71.59%)	477,917 (55.79%)	
Other/Multiple	283,785 (2.46%)	156,558 (2.12%)	36,613 (1.90%)	43,637 (3.13%)	46,977 (5.48%)	
Missing	12,798 (0.11%)	1,866 (0.03%)	6,018 (0.31%)	3,736 (0.27%)	1,178 (0.14%)	
Maternal Education Category						<0.001
Missing	311,940 (2.70%)	272,028 (3.69%)	22,313 (1.16%)	8,350 (0.60%)	9,249 (1.08%)	
No HS	615,539 (5.33%)	525,564 (7.13%)	42,555 (2.21%)	19,731 (1.42%)	27,689 (3.23%)	
Some HS	1,600,321 (13.86%)	1,093,053 (14.83%)	222,220 (11.55%)	152,621 (10.95%)	132,427 (15.46%)	
HS Degree	2,918,615 (25.28%)	1,813,146 (24.61%)	519,456 (26.99%)	372,219 (26.71%)	213,794 (24.96%)	
Some College	2,943,517 (25.50%)	1,716,359 (23.29%)	571,385 (29.69%)	384,055 (27.56%)	271,718 (31.72%)	
4 Yr College	1,990,099 (17.24%)	1,226,607 (16.65%)	344,614 (17.91%)	286,768 (20.58%)	132,110 (15.42%)	
>4 Yrs College	1,163,345 (10.08%)	722,119 (9.80%)	201,775 (10.49%)	169,843 (12.19%)	69,608 (8.13%)	
Maternal Payor Category						<0.001
Missing	1,345 (0.01%)	897 (0.01%)	7 (0.00%)	158 (0.01%)	283 (0.03%)	
Private	5,798,266 (50.23%)	3,591,150 (48.73%)	1,015,234 (52.76%)	829,713 (59.54%)	362,169 (42.28%)	
Government	5,371,879 (46.54%)	3,555,407 (48.25%)	799,841 (41.56%)	548,729 (39.38%)	467,902 (54.62%)	

© 2023 Kozhimannil KB et al. *JAMA Health Forum.*

Self Pay	247,641 (2.15%)	197,453 (2.68%)	12,670 (0.66%)	14,756 (1.06%)	22,762 (2.66%)	
Other	124,245 (1.08%)	23,969 (0.33%)	96,566 (5.02%)	231 (0.02%)	3,479 (0.41%)	
Comorbidity Count	0.81 (1.06)	0.75 (1.02)	0.95 (1.17)	0.84 (1.05)	0.94 (1.14)	<0.001
Comorbidity score for SMM	3.84 (6.32)	3.47 (5.97)	4.66 (7.05)	4.14 (6.44)	4.79 (7.02)	<0.001
Comorbidity score for non-transfusion SMM	4.77 (8.52)	4.31 (8.05)	5.77 (9.47)	5.19 (8.70)	5.83 (9.44)	<0.001
High Risk: Comorbidity Count >=1						<0.001
Low Risk	5,797,093 (50.22%)	3,871,103 (52.53%)	870,063 (45.21%)	670,614 (48.12%)	385,313 (44.98%)	
1 or more comorbidities	5,746,283 (49.78%)	3,497,773 (47.47%)	1,054,255 (54.79%)	722,973 (51.88%)	471,282 (55.02%)	

© 2023 Kozhimannil KB et al. *JAMA Health Forum.*
