# Supplementary material for: Obstetric Volume and Severe Maternal Morbidity Among Low-Risk and Higher-Risk Patients Giving Birth at Rural and Urban US Hospitals
Source: JAMA Health Forum. 2023 Jun 24;4(6):e232110. doi: 10.1001/jamahealthforum.2023.2110 (PMC10290751; doi:10.1001/jamahealthforum.2023.2110)
Supplement: Supplement 2. — Data Sharing Statement [file jamahealthforum-e232110-s002.pdf]

Data Sharing Statement

Kozhimannil. Obstetric Volume and Severe Maternal Morbidity Among Low-risk and Higher-risk Patients Giving Birth at Rural and Urban US Hospitals. *JAMA Health Forum*. Published June 24, 2023. doi:10.1001/jamahealthforum.2023.2110

Data
Data available: No

Additional Information
Explanation for why data not available: Data used in this analysis contain identifiable personal health information and were obtained through data use agreements with each state that prohibit any sharing of these data. Others wanting access to these data would need to obtain their own data use agreements with the individual state departments of health.
